# The Relationship Between Cognitive Decline and Sedentary Time

**DOI:** 10.1093/geroni/igaa057.618

**Published:** 2020-12-16

**Authors:** Lorraine Phillips, Mary Bowen

**Affiliations:** University of Delaware, Newark, Delaware, United States

## Abstract

Early identification of functional decline in older adults with mild cognitive impairment (MCI) provides the opportunity to initiate behavioral interventions to slow decline. More frequent breaks in sedentary time has been associated with greater lower extremity function. This longitudinal study examined the effect of 6-month change in cognitive function on monthly sedentary time, controlling for lower extremity function, among community-dwelling older adults with MCI. Twenty adults with Montreal Cognitive Assessment Score (MoCA) between 19-25, who were age ≥ 60 years old, and ambulatory, wore an actigraph for 6 months and participated in monthly in-person assessments. Measures included MoCA change (baseline to month 6), Short Physical Performance Battery (SPPB; baseline, months 3 and 6); sedentary time and physical activity intensity; and falls (monthly). The sample was 70% female, 60% non-Hispanic white, with a mean age of 77 years. Sixteen participants provided complete data for mixed-model analysis. Over 6 months, 11 falls occurred among 7 participants. The mean MoCA score declined from 22.7 to 21.9 while SPPB remained stable. Overall time spent in sedentary behavior was high (71%) and physical activity intensity was low (light and moderate combined= 26.1%). Results of multi-level analysis with sedentary time as a continuous Level-1 variable and MoCA change scores, SPPB scores, and age in Level-2 showed that negative change in MoCA (β=-0.11; p≤0.05) was associated with increased sedentary time. Given sedentary time increases as cognitive function declines, older adults with MCI could benefit from interventions designed to interrupt sedentary time as well as increase physical activity.

